# Influenza Complications in Children: The Experience of a Children’s Hospital in Romania and a Comparative Literature Review of Western and Eastern Studies

**DOI:** 10.3390/diseases14070229

**Published:** 2026-06-26

**Authors:** Ioana Luca, Laura Bleotu, Oana Gabriela Falup-Pecurariu

**Affiliations:** 1 Faculty of Medicine, Transilvania University, 500036 Brasov, Romania; 2 Department of Pediatrics, Children’s Clinical Hospital, 500063 Brasov, Romania

**Keywords:** influenza, children, complications, hospital admission length, cost

## Abstract

Background: Influenza infections have reached an approximate number of one billion cases annually in the general population. Hospitalization due to this infection is associated with high morbidity, and a proportion of hospitalized children may require ICU admission. In the United States of America, one in five children hospitalized due to influenza requires transfer to the intensive care unit (ICU). The real burden of this disease is not accurately known, especially for the pediatric population. Objective: The objective of this study was to define the characteristics of influenza-associated complications in pediatric patients hospitalized at a tertiary hospital in Brasov, Romania. Methods: This was an observational, retrospective study that gathered 258 influenza-infected patients aged from 0 up to 18 years old, hospitalized during the period from 1 January 2020 to 31 December 2025 at the Children’s Hospital of Brasov (a single-center study, but in a tertiary unit). The complications from this disease were categorized into respiratory, hematological, musculoskeletal, renal, ENT, cutaneous, rheumatological, and bacterial superinfections. Results: The patients were stratified according to their influenza type (A or B) and length of hospital stay. The length of stay was categorized as 0–4 days, 5–10 days, or >10 days. No significant association was observed between the influenza type and admission duration (χ^2^ = 2.185, df = 2, *p* = 0.3354). The most frequent complications were respiratory—bronchiolitis and pneumonia (22.8%)—followed by hematological (13.5%). Conclusions: The length of stay did not differ significantly between patients with influenza A and those with influenza B in the selected sample. The most common complications were respiratory, hematological, ENT, and neurological.

## 1. Introduction

The influenza virus belongs to the Orthomyxoviridae family, with three types (A, B, and C), but only types A and B are responsible for seasonal epidemics. These specific types are divided into subtypes (H1N1 and H3N2) for type A, while type B is divided into the Victoria and Yamagata lineages [1].

Influenza symptoms and complications in the pediatric population are more diverse than those in the adult population, ranging from gastrointestinal manifestations (diarrhea, vomiting) and marked apathy to well-known respiratory components (cough, respiratory distress) and hematological, renal, or neurological damage [2].

The complexity of the clinical involvement translates into possible multivisceral involvement; the medical literature describes hematological complications (cytopenias—anemias, leukopenias, thrombocytopenias, coagulation disorders) [3], renal involvement [4] (although rather rare—cases of acute renal failure or hemolytic uremic syndrome, secondary to influenza infections), and neurologic suffering (encephalopathies, seizures, encephalitis) [5], all these being lesser-known facets of this disease.

Furthermore, the influenza virus creates a suitable environment for bacterial growth by altering the response of the immune system and disrupting the integrity of mucosal barriers, leading to bacterial superinfections or co-infections after influenza, which may result in high mortality levels in hospitalized patients [6,7,8].

According to American data, almost 10% of all pediatric hospitalizations in the United States are secondary to influenza infection [9], accounting for up to 700,000 hospitalizations [10], with a global hospitalization rate of approximately 1 million solely for children under the age of 5 [11].

Internationally, the burden of influenza is unknown, especially for the pediatric population, but estimated numbers reach a billion cases of influenza per year, with 650,000 deaths annually in the general population. Although the severity of influenza is different by year, because of the circulating main strain or new mutations that may occur, there are rising concerns about a higher number of influenza cases and higher complication rates [12].

Therefore, accurate data are needed—particularly for the pediatric age group, which may face higher complication rates, and particularly for Romania, where the burden of influenza in children is widely unspecified due to the scarcity of specific literature on this subject.

This subject is even more important in a country where the influenza vaccination rate in the general population for the last season was 6% and that in the pediatric population was 3%.

The data from Brasov County on influenza-vaccinated pediatric residents situate the rate at approximately 4.60% of all representatives of the 0–18-year-old age group (from 1 September 2025 until 22 January 2026). In comparison, the highest-coverage influenza vaccination rates were in Spain (48.2%) for children aged 6 months to 5 years old and Finland (32.9%) for the same age group. Lithuania and Poland report rates similar to the Romanian one, ranging from 2 to 4% [13].

The aim of this study is to evaluate the course of influenza-infected patients in our hospital, with the first research objective focused on epidemiological trends in our area (pandemic and post-pandemic) and a second research objective of emphasizing a possible scientific gap for this pathology and its complications in the region of Central and Eastern Europe.

## 2. Materials and Methods

We present an observational, retrospective study which gathered 258 patients aged between 0 and 18 years old, hospitalized during the period from 1 January 2020 to 31 December 2025 at the Children’s Hospital of Brasov (a single-center study). All of them had a positive rapid antigen influenza A or B test at admission. The utilized tests were lateral flow chromatographic immunoassay qualitative tests that detect SARS-CoV-2, respiratory syncytial virus, and influenza A and B from nasal and naso-oropharyngeal swab (Goldsite Diagnostics Inc., Shenzhen, China). Although one may discuss the absence of PCR confirmation, we consider it to not have induced misclassification bias, as the rapid antigen tests that we used allowed for rapid, clear, and easy testing of all children who presented in our Emergency Department with influenza-like illness. The characteristics of the rapid antigen test kits are as follows [14]:

- Sensitivity for influenza type A: 95.0%;
- Specificity for influenza type A: 99.1%;
- Accuracy for influenza type A: 98.4%;
- Sensitivity for influenza type B: 92.9%;
- Specificity for influenza type B: 99.1%;
- Accuracy for influenza type B: 98.1%.

(SARS-CoV-2 & Flu A/B & RSV Combo Antigen Test, Goldsite Diagnostics Inc., Shenzhen, China)

We focused on the patients’ clinical evolution, epidemiological and demographical characteristics, laboratory test abnormalities, influenza-associated complications, and comorbidities, as well as the financial aspects of these hospitalizations.

All included children met the case definition of an influenza-like illness (ILI), and presented with:

- Fever;
- And/or nasal discharge;
- And/or sneezing;
- And/or dry cough;
- And/or respiratory distress;
- And/or malaise;
- And/or digestive issues, such as diarrhea, nausea, or vomiting.

We excluded cases where influenza was accidentally found, secondary to our hospital virologic screening (trauma admissions or day-care service). The presented study was conducted respecting the principles of the Declaration of Helsinki, and the legal guardians of all participants offered their written informed consent. The Ethics Committee of Brasov Children’s Hospital approved the study (approval code: 9/31026, approval date: 16 February 2026). The aim of our study was to define the characteristics of influenza-associated complications in pediatric patients hospitalized at a tertiary hospital in Brasov, Romania.

The patients were stratified according to their influenza type (A or B) and length of hospital stay. The length of stay was categorized as 0–4 days, 5–10 days, or >10 days. Data are presented as absolute numbers and percentages. One patient requested to leave earlier than advised; we did not include him in the table, because it would affect the statistics.

The patients were also stratified according to their month of admission and length of hospital stay. The length of stay was categorized as above, and data were summarized as absolute numbers and percentages. The same patient who requested to leave earlier than advised was excluded from the table. The study population was stratified again by gender and age group (0–<1, 1–5, 6–12, and 13–18 years). A χ^2^ (chi-square) statistical comparison test was performed on all contingency tables (Tables 3–6) in order to determine whether there was any statistical correspondence between the analyzed criteria. The statistical analysis was performed using GraphPad Prism version 10.2.3 (GraphPad Software, Boston, MA, USA).

Complications were grouped into neurological, hematological, rheumatological, renal, ENT, musculoskeletal, tegumentary, and bacterial superinfections.

Neurological symptomatology was defined as febrile or afebrile seizures, or bulging of the fontanelle; respiratory involvement was considered in every child who presented with a level of oxygen saturation under 94%, costal draft, wheezing, or oral cyanosis, as well as those with radiographically confirmed pneumonia.

For hematological complications, we included all cases of thrombocytopenia (either isolated or in immune thrombocytopenic purpura secondary to influenza infection; thrombocyte values <150.000/mm^3^), newly instituted neutropenia (leukocyte values <4.500/mm^3^), or hemolytic anemia associated with influenza infection. Rheumatological involvement was considered in cases of influenza-associated vasculitis or arthritis, while renal complications were highlighted by relapses of previously diagnosed nephrotic syndrome, or acute glomerulonephritis after influenza infection. The other categories of influenza-associated complications were ENT infections (catarrhal otitis media or purulent otitis media), musculoskeletal (myositis), cutaneous involvement (rashes and purpura), and bacterial superinfections.

The comorbidities reported in the study population could be grouped as neurologic disorders (epilepsy, cerebral palsy, microcephaly), cardiovascular disorders (arterial hypertension, atrial septal defect, congenital heart disease, Wolff–Parkinson–White syndrome), endocrine and metabolic diseases (type 1 diabetes mellitus), hematological disorders (beta-thalassemia), genetic syndromes (Neurofibromatosis type 1, Phelan–McDermid syndrome), and renal illness (nephrotic syndrome).

## 3. Results

The baseline characteristics of the study population are presented in Table 1.

A total of 258 patients were included, with 44 cases in 2020, 0 in 2021, 94 in 2022, 11 in 2023, 50 in 2024, and 59 in 2025. Neurological symptomatology was defined as febrile or afebrile seizures, or bulging of the fontanelle; respiratory involvement was considered in every child that required oxygen supplementation (level of oxygen saturation under 94%, costal draft, wheezing, or oral cyanosis), as well as those with radiographically confirmed pneumonia.

For hematological complications, we included all cases of thrombocytopenia (either isolated or in immune thrombocytopenic purpura secondary to influenza infection; thrombocyte values <150.000/mm^3^), newly instituted neutropenia (leukocyte values <4.500/mm^3^), or hemolytic anemia associated with influenza infection. Rheumatological involvement was considered in cases of influenza-associated vasculitis or arthritis, while renal suffering was highlighted by relapses of previously diagnosed nephrotic syndrome, or acute glomerulonephritis after influenza infection. The other categories of influenza-associated complications consisted of ENT infections (catarrhal otitis media or purulent otitis media), musculoskeletal (myositis), cutaneous involvement (rashes and purpura), and bacterial superinfections, as detailed in Table 2. We also mention one case of incomplete Kawasaki syndrome in the studied population, but the child had both influenza and a rotavirus infection; therefore, it was difficult to incriminate one of the aforementioned infectious triggers.

Treatment was also noted for all studied patients, with special attention given to antivirals (oseltamivir), antibiotics, oxygen supplementation, and cortisone (dexamethasone, methylprednisolone, prednisone, or hemisuccinate hydrocortisone). Financial aspects were covered by checking the hospitalization costs of each patient. The geographical provenance of the studied children was noted as U for urban areas and R for rural ones, and the months of hospital admission, as well as the type of influenza (A or B), were also included.

The population stratification is detailed in Table 3, Table 4, Table 5 and Table 6 (for age group, gender, type of influenza, length of stay, and complications).

The patients were stratified according to their influenza type (A or B) and length of hospital stay (Table 3). The length of stay was categorized as 0–4 days, 5–10 days, or >10 days. Data are presented as absolute numbers and percentages. 

No significant association was observed between the influenza type and admission duration (χ^2^ = 2.185, df = 2, *p* = 0.3354). Length of stay did not differ significantly between patients with influenza A and those with influenza B in this sample.

The patients were then stratified according to their month of admission and length of hospital stay (Table 4). The length of stay was categorized as above, and the data were summarized as absolute numbers and percentages. 

The chi-square statistic was 11.4326, and the *p*-value was 0.17837. The result was not significant at *p* < 0.05, so there was no clear association between the month of hospital admission and the hospital admission duration in our studied sample.

The study population was then stratified by gender and age group (0–<1, 1–5, 6–<13, and 13–18 years) (Table 5). Male patients predominated in the 0–12 y.o age group, while female patients were more frequent in the teenage group (13–18 years old). Although the number of hospitalized adolescent girls was almost double that of boys (13 girls vs. 7 boys), the adolescent sample was quite small (n = 20), so this difference could have occurred by chance. Based on the studied population and a *p*-value of 0.263, there is insufficient evidence to conclude that adolescent girls were hospitalized more often than adolescent boys in our studied population.

A χ^2^ (chi-square) statistical comparison test was performed on the population stratified by age group and gender to determine whether the gender distribution varied across the age groups.

No significant association was observed between age group and gender (χ^2^ = 3.967, df = 8, *p* = 0.8601).

In Table 6, stratification of the population by age group (<1 year old, 1–5, 6–< 13, 13–18 years old) and complications is presented; a chi-square test determined that there was no statistically significant association between the age group and the occurrence of complications (χ^2^ = 6.49, *p* = 0.09).

**Figure 1:**
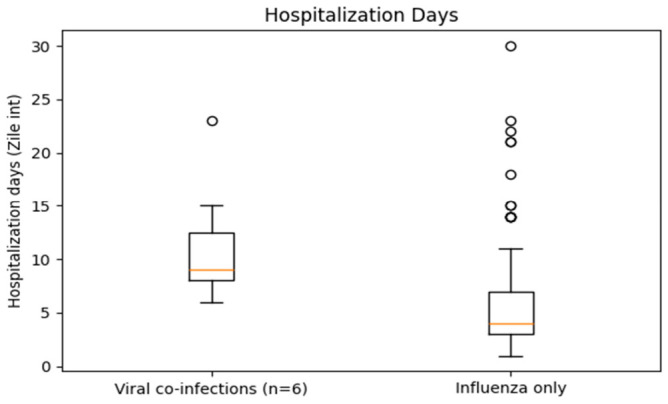
Boxplot describing the number of hospitalization days in influenza-only patients versus patients with viral co-infections.

There were six patients with reported viral co-infections (either influenza A + B, influenza + respiratory syncytial virus, or influenza + SARS-CoV-2), all of them diagnosed via rapid antigen tests. In the studied population, patients with viral co-infections had significantly longer hospital stays than those without viral co-infection (median 9 vs. 4 days, *p* = 0.002) (Figure 1).

As to the incidence of complications, it was rather difficult to evaluate whether there was a higher risk of incidence in the patients with viral co-infections compared to those with an isolated influenza infection; statistical significance was not reached (*p* = 0.70) for the studied population, although complications were more frequent in the viral co-infection subgroup (66.7% of patients with viral co-infections compared with 55.7% of patients without viral co-infections).

Therefore, viral co-infections were associated with a longer hospital stay (Figure 1), but not with a significantly higher risk of complications in this studied group.

It is important to note that the number of admissions in 2023 may seem rather low (11 hospital admissions), but the percentage of hospital admissions after a positive antigen test remained in the 3–5.54% range that was observed in all studied years, with a peak in 2022 (Table 7).

The complications associated with influenza infection in the studied population were classified by the affected anatomical structure into respiratory, rheumatological, hematological, renal, neurological, ENT, mucocutaneous, and bacterial superinfections, as shown in Table 2.

In the analyzed group (n = 258), the most frequent complications were respiratory—bronchiolitis and pneumonia—followed by hematological manifestations, bacterial superinfection, and neurological manifestations. Among the studied population, 152 patients (58.9%) presented at least one complication, and multiple complications were noted in 13 children (5% of the population)—specifically, associations of respiratory complications with rashes or ENT manifestations.

Leukopenia was noted in 11.6% of our studied population (30 children out of 257 had leukocyte values under the threshold of 5.000/µL).

**Figure 2:**
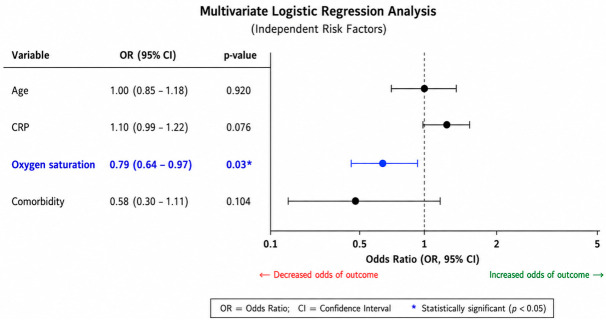
Independent risk factors associated with odds of complications. The dashed line at OR = 1 is the null value (or line of no effect) in the figure.

Among the 258 hospitalized children, 152 (58.9%) developed at least one complication. Patients with complications had higher CRP levels (median 0.70 vs. 0.33 mg/dL, *p* < 0.001) and lower oxygen saturation at admission (95% vs. 98%, *p* < 0.001). No significant associations were observed for age, sex, or comorbidities. In a multivariable logistic regression analysis, lower oxygen saturation remained independently associated with complications (OR 0.79, *p* = 0.03). The multivariate regression analysis to identify independent risk factors for major complications is summarized in Table 8 and Figure 2.

The cost of hospitalization for the studied population ranged from 30 to 63.000 RON (6.94 to 14.574 USD), with an average of 4869.95 RON/patient (1126 USD).

Treatment-wise, only seven patients out of 258 were not administered oseltamivir (2.7%) as an antiviral treatment for influenza infection, so the sample was not large enough to compare the children who received antiviral treatment and those who did not receive oseltamivir in terms of their clinical evolution.

Nine children (3.5% of the studied population) presented with severe respiratory failure and needed ICU admission. Of them, eight children (88.9%) were from the 0–2 year age group. Almost half of the patients admitted to the ICU (44.4%) had associated bacterial or viral co-infections (*Streptococcus pneumoniae*, *Mycoplasma pneumoniae*, syncytial respiratory virus, SARS-CoV-2). Two children required invasive mechanical ventilation, and one of them succumbed to multi-organ failure. Only one child was known to have neurological-associated comorbidities, while the others were considered to be immunocompetent.

## 4. Discussion

Data on influenza complications in children from Eastern Europe remain rather scarce, but in other retrospective studies from Romania and Poland, like in our study, respiratory complications were the most prevalent in the studied populations [15,16,17,18]. All four selected studies found that the pre-school age group was the most affected, with a prevalence of type A influenza, which is consistent with the findings of our study. The median ages in the study from Bucharest and the Polish study were similar to that in our findings (4.4 and 4.7, compared to 5.3 in our study) [15,16]. Our findings also align with the other Eastern European single-center retrospective studies in reporting a majority of respiratory, hematological, or ENT complications post-influenza infection [17,18].

Table 9 and Table 10 highlight the main studies on influenza complications in children from the Western world (Western Europe/United States of America) and those from Central and Eastern Europe.

### 4.1. Neurological Complications

The French and American literature on neurological complications of influenza infection in pre-pandemic and post-pandemic hospitalizations shows similar outcomes to our study (a range from 7 to 10%, compared to 10.8% in our study), with the majority of neurological complications being febrile and non-febrile seizures with a rather favorable evolution [24,25]. Differences stem from the fact that none of the children in our study developed influenza-associated encephalopathies or encephalitis but, even in the presented studies, the number of severe complications was rather low. There were zero cases of Guillain–Barré syndrome, meningitis, or stroke in our studied population.

Interestingly, there are few accounts of bulging fontanelle associated with type A influenza infection; the infant in our study did not present any other neurological dysfunctions, and the anterior fontanelle bulging was transitory and did not leave any sequelae. Although studies do associate this clinical manifestation with febrile viral infections in some cases, it is more commonly associated with SARS-CoV-2, rotavirus, and exanthema subitum cases [26,27,28].

### 4.2. ENT and Bacterial Superinfections

The occurrence of ENT complications and bacterial superinfections in our study is consistent with findings from the international literature on this subject, with multiple accounts of otitis media and rhinopharyngitis (10 to 50% of children develop these complications after influenza infection—16.2% in the presented study) [29].

The most common etiological agent of secondary pneumonia after influenza infection was *Streptococcus pneumoniae* (13% of all bacterial superinfections from the studied population), followed by *Haemophilus influenzae* and methicillin-sensitive *Staphylococcus aureus*. This is in agreement with a medical literature review on the subject, where *S. pneumoniae* is described as the most frequent culprit of post-influenza bacterial pneumonia [30].

In the studied population, only 11.6% of the children presented with leukopenia. This may be a particularity of our studied population, and it could also be correlated with the relatively high proportion of viro-bacterial associations in the studied group, as shown in Table 2.

### 4.3. ICU Admissions

The selected Western [19,20,21,22,23] studies highlight the main complication categories of respiratory complications, which are the most common and the leading cause of hospitalization; neurological complications, in which seizures are the predominant manifestation; and systemic complications—sepsis, multi-organ failure, and ICU admission—especially in the 0–2 years age group, the unvaccinated, and those with associated comorbidities such as neurologic/cardiovascular diseases or malnutrition.

These findings are consistent with the findings from our study group, as respiratory and neurological complications prevailed. The ICU admissions from these selected studies were from the infant and toddler age group, similar to those in our study. An interesting difference stems from the fact that only one child from our study population who required ICU admission had neurological comorbidities, while the others were considered to be immunocompetent. The American and Western European studies highlight a higher prevalence of severe influenza complications in children in association with other chronic diseases.

### 4.4. Renal Complications

As presented in Table 2, five children from the studied population presented with renal suffering (four of them had chronic nephrotic syndrome and presented with relapses associated with influenza infection; the fifth child had intra-infectious acute glomerulonephritis). Acute renal injury is a rather common complication seen in adults with severe influenza admitted to the ICU [31]. However, the data in children are insufficient, and these types of complications are associated in pediatric patients with previously diagnosed kidney comorbidities or children experiencing multiorgan dysfunction syndrome. The majority of the literature comes from the experience of the 2009 H1N1 influenza pandemic, where acute kidney injury, acute tubular necrosis, hemolytic uremic syndrome, and rhabdomyolysis were described.

Cases of intra-infectious acute glomerulonephritis and hematuria associated with influenza infection are described in American and Italian studies, and they are consistent with the findings from our study, as type A influenza was responsible for the acute renal suffering in the studied population. These manifestations may be explained by direct renal injury, generalized inflammation, or secondary microthrombi that affect kidney vascularization [32,33,34,35,36,37].

### 4.5. Skin and Muscle Complications

In the studied population, there were two cases of intra-infectious myositis (only 0.8% of all included cases), which is consistent with the findings in the literature. Although this complication is, rather, associated with type B influenza, both types of influenza were equally represented in our sample (one patient with myositis had type A infection, and the other one tested positive for type B) [38].

Cutaneous manifestations from influenza infection, such as petechiae or maculo-papular rashes associated with viral infections, are actually quite well-documented, although they are rather rare [39]. In the studied population, there were six such cases (four of them with transient generalized macular or papular rashes, one with intra-infectious vasculitis, and one with incomplete Kawasaki syndrome).

The main trigger of Kawasaki disease has not yet been elucidated, with infectious agents being one of the suspected causes. The literature describes several case reports, specifically type A influenza (H1N1) [40]. This may be consistent with the findings from our studied population, as the child with incomplete Kawasaki syndrome had type A influenza, but we cannot state that this was the real culprit as he presented with a concomitant rotavirus infection.

Purpuric rash and vascular involvement represent other possible rare manifestations that may arise in an infection with the influenza virus; the clinician must remain vigilant and exclude other severe causes of petechiae, such as purpura fulminans or severe coagulation disorders. The one case from our studied population was classified as leukocytoclastic vasculitis, most probably caused by a type B influenza virus infection. The literature states that the most common cause of this manifestation is a streptococcal infection [41]. Some cases of leukocytoclastic vasculitis have been associated with influenza vaccination in adults [42], suggesting that there may be some degree of generalized inflammation or autoimmunity activation, but these cases are rather rare and do not diminish the benefits of, or the compelling need for, influenza vaccination for prophylaxis.

There have been a few cases of influenza-associated leukocytoclastic vasculitis in children, but a Korean case report [43] describes this manifestation in a two-year-old child who was infected with type A influenza. This is inconsistent with the findings from our studied population, where type B influenza was the main culprit, but these results are important, as they signal that influenza complications in children may be wider than previously thought.

### 4.6. Other Aspects of Influenza Infection and Limitations of This Study

The epidemiology of influenza in the studied population was affected by the SARS-CoV-2 pandemic, as the cohort spanned from 2020 to 2025.

**Figure 3:**
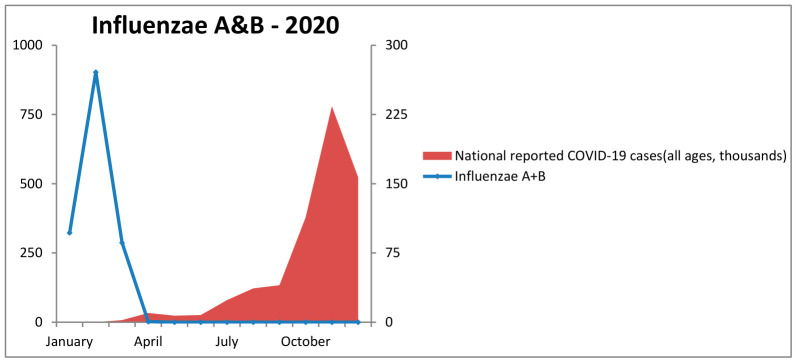
National reported COVID-19 cases (all ages) and Influenza cases from Children Hospital of Brasov in 2020.

**Figure 4:**
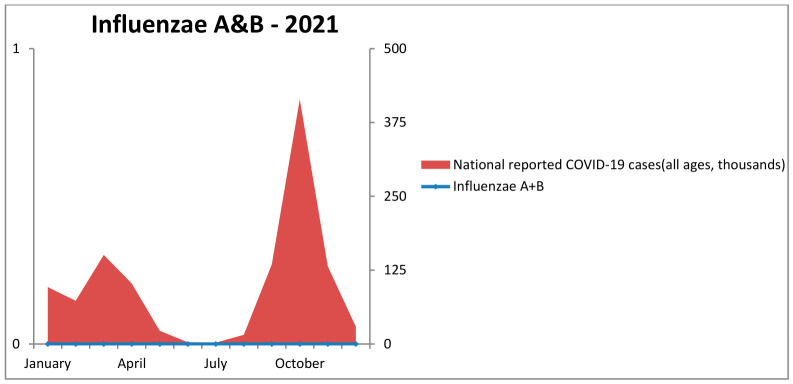
National reported COVID-19 cases (all ages) and Influenza cases from Children Hospital of Brasov in 2021.

**Figure 5:**
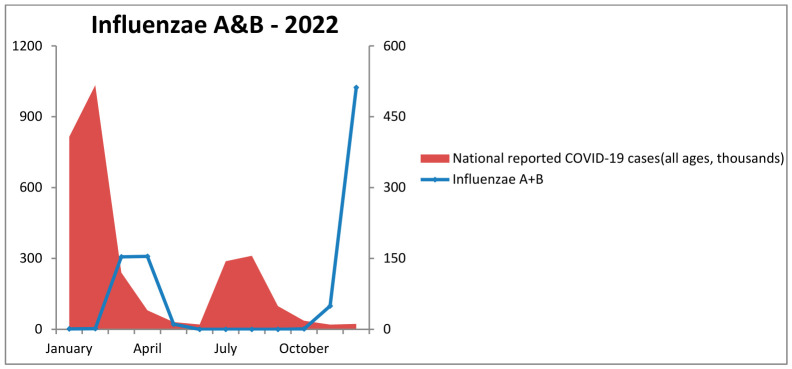
National reported COVID-19 cases (all ages) and Influenza cases from Children Hospital of Brasov in 2022.

Figure 3, Figure 4 and Figure 5 show a decrease in influenza cases in the second part of 2020 (quarantine measures in Romania were implemented starting from March of 2020) and all of 2021. The number of COVID-19-positive tests simultaneously began to grow rapidly in the second part of 2020, and the SARS-CoV-2 virus was present during all of 2021 (with a decrease in June and July 2021). In 2022, the peak number of SARS-CoV-2-infected patients occurred in February, and there was a descending slope afterwards, while influenza cases were most numerous in December 2022.

Our study suggests that there was a decrease in influenza cases in the second part of 2020 and during all of 2021 (*p* = 0.029). The highest number of influenza cases among all these observed years was reported in 2022, when the majority of COVID-19-associated restrictions were abolished. It is important to mention that, despite the discussion about co-infections, the international trend of all-time-low numbers of registered cases of other viral infections during the pandemic peak is consistent with the trend presented in our study: in Australia, influenza infection cases were reduced by 98–99.4% compared to other seasons (2012–2019), with similar numbers being reported in Chile, South Africa, Spain, and the United States of America [44]. Non-pharmaceutical measures that were implemented in Romania starting from March of 2020 (mask wearing, social distancing) could have played an important role in the decrease in influenza cases that was observed from the second part of 2020 and during all of 2021. The highest number of influenza cases among all these observed years was reported in 2022, when the majority of restrictions were abolished and children were left with an “immunity gap” [45] after almost 2 years of reduced exposure to viral or bacterial agents. Our data are consistent with the international trends.

The testing policies in our tertiary care unit were stable during the presented years, and all children who presented in the Emergency Department with influenza-like illness symptoms were tested for influenza with a rapid antigen test.

The percentage of hospital admissions after a positive antigen test remained in the 3–5.54% range that was observed in all studied years, with a peak in 2022.

Seven patients from the studied cohort did not receive antiviral treatment. The national guidelines recommend early administration of neuraminidase inhibitors [46] (oseltamivir), specifically in the first 24–48 h after symptoms appear. The attending physicians considered oseltamivir to not be needed in the case of these seven patients, as they presented to the Emergency Room more than 2 days after their symptoms began.

The financial aspect of the presented study revealed that approximately 1155 EUR is spent for each influenza-infected hospitalized patient. A literature review that focused on the cost of influenza-associated hospitalization in European children (Romania was not included) [47] presented a range of 74 to 252 EUR per patient. It is important to mention that we did not take into account secondary costs, like unpaid leave for the parents of children who required hospital admission, which may raise the sum to even higher numbers. It is also necessary to mention that the vast majority of literature reviews about the cost of influenza hospitalizations discuss pre-pandemic hospital admissions, with little data about the evolution of post-pandemic admissions.

The costs of hospitalization in our study are relatively high compared to the European average costs, although studies in this area are scarce. A possible explanation for these elevated costs in our studied population may be that almost half of the studied children had hospitalizations longer than 5 days (49.4%), and 108 patients received antibiotics (42%). Therefore, antibiotic administration and prolonged hospital stays result in a high financial burden.

The limitations of this study result from this being a single-center study (although in a tertiary hospital), as well as not being able to provide the serotypes of the involved influenza strains.

The studied population was composed of all the patients who met the inclusion and exclusion criteria; therefore, it is important to mention that it was a heterogeneous population, as our unit is a tertiary care hospital. The inclusion of children with known comorbidities along with supposedly immunocompetent and previously healthy children may have affected the validity of the reported complication rates in this study.

It is important to mention that even though the rapid antigen tests we used in this study for the diagnosis of influenza allowed for clear, easy screening in children presenting with influenza-like illness symptoms, the lack of subsequent PCR confirmation may have led to a small proportion of undiagnosed cases, as the sensitivity of the test was not 100%.

Despite the presented limitations, this study is important because it recognizes the potential severity and complexity of influenza complications, specifically in the pediatric population, with special attention drawn to a rather geographically challenged area in Eastern Europe, for which medical data may be scarce.

Interestingly, Table 9, which details five of the most important Western studies on this subject [19,20,21,22,23]—multicentric, detailed studies—highlights severe influenza complications in children, while the four studies [15,16,17,18] from Central and Eastern Europe detailed in Table 10 are more oriented towards the epidemiological aspect and on statistical analysis and do not reveal such dramatic outcomes in influenza-infected children. This does not mean that severe cases of influenza/deaths/sequelae do not happen in this area of the world, but it may be a matter of underreported data.

Finally, these differences between Eastern and Western Europe may be secondary to discrepancies in vaccination rates, hospitalization criteria, clinical and therapeutic approaches, and discharge and follow-up criteria, highlighting the need for specific and standardized guidelines, along with epidemiological surveillance and the completion of multiple scientific gaps.

## 5. Conclusions

In the presented study, the length of stay did not differ significantly between patients with influenza A and those with influenza B in the selected sample. No significant association was observed between the age group and gender distribution, and no association was found between the age group and the risk of developing complications during or after influenza infection in the studied population.

The most common complications were respiratory, hematological, ENT, and neurological. Viral co-infections were associated with a longer hospital stay, but not with a significantly higher risk of complications in this studied group. Patients with complications had higher CRP levels and lower oxygen saturation at admission.

The literature from Eastern and Central Europe on this subject is rather scarce, highlighting a possible scientific gap.

## Figures and Tables

**Table 1:** Baseline characteristics of the study population.

Characteristic	Overall Population (n = 258)
Age, years (mean ± SD)	5.3 ± 4.2
Sex, male, n (%)	145 (56.2)
Sex, female, n (%)	113 (43.8)
Length of hospital stay, days (median [IQR])	4.0 [3.0–7.0]
C-reactive protein (mean ± SD)	2.2 ± 4.2
Hemoglobin (mean ± SD)	11.9 ± 1.8
Leukocytes (mean ± SD)	10.0 ± 5.7
Platelets (mean ± SD)	304.2 ± 143.5
Oxygen saturation %, mean ± SD	96.3 ± 3.3
Oseltamivir (Tamiflu), n (%)	248 (96.1)
Antibiotics, n (%)	108 (41.8)
Any comorbidity, n (%)	92 (35.6)
Any complication, n (%)	152 (58.9)

Data are presented as mean ± standard deviation, median [interquartile range], or number (percentage), as appropriate.

**Table 2:** Complications observed in the study population.

Complication	n (%)
Hematological—thrombocytopenia	21 (8.1)
Hematological—leukopenia	13(5)
Renal	5 (1.93)
ENT	16 (6.2)
Tegumentary (rash)	5 (1.93)
Rheumatological—vasculitis	1 (0.38)
Rheumatological—arthritis	2 (0.77)
Musculoskeletal—myositis	2 (0.77)
Bacterial superinfection	30 (11.6)
Neurological	28 (10.8)
Respiratory	59 (22.8)

Data are presented as the number (percentage) of patients. Percentages were calculated from the total study population. One patient may have experienced several complications simultaneously.

**Table 3:** Stratification of patients by influenza type and length of stay (*p* = 0.3354).

	Influenza A	Influenza B	Total
0–4 days n (%)	93 (53.4%)	37 (44.6%)	130 (50.6%)
5–10 days n (%)	69 (39.6%)	41 (49.4%)	110 (42.8%)
>10 days n (%)	12 (7%)	5 (6%)	17 (6.6%)
Total n (%)	174 (100%)	83 (100%)	257 (100%)

n = number of patients; % = percentage within each month (row-wise). The last row (“Total”) shows the overall length of admission in the study population.

**Table 4:** Stratification of hospital admissions by month and length of stay (*p* = 0.17837).

Month of Admission	0–4 Days n (%)	5–10 Days n (%)	>10 Days n (%)	Total n (%)
January	22 (43.1%)	25 (49%)	4 (7.9%)	51 (100%)
February	25 (51%)	21 (42.8%)	3 (6.2%)	49 (100%)
March	46 (67.6%)	19 (27.9%)	3 (4.4%)	68 (100%)
April	17 (44.7%)	19 (50%)	2 (5.3%)	38 (100%)
May	0 (0%)	3 (100%)	0 (0%)	3 (100%)
June	0 (0%)	0 (0%)	1 (100%)	1 (100%)
July	0 (%)	0 (%)	0 (0%)	0 (%)
August	0 (%)	0 (%)	0 (0%)	0 (%)
September	0 (%)	0 (%)	0 (0%)	0 (%)
October	0 (%)	0 (%)	0 (0%)	0 (%)
November	1 (100%)	0 (0%)	0 (0%)	1 (100%)
December	19 (41.3%)	24 (52.2%)	3 (6.5%)	46 (100%)
Total	130 (50.5%)	111 (43.2%)	16 (6.3%)	257 (100%)

n = number of patients; % = percentage within each month (row-wise). The last row (“Total”) shows overall length of admission in the study population.

**Table 5:** Population stratification by gender and age group in the studied population (*p* = 0.8601).

Age Group (Years)	Male, n (%); Female, n (%)	Total, n (%)
0–<1	41 (57.7%); 30 (42.3%)	71 (100%)
1–5	66 (57.9%); 48 (42.1%)	114 (100%)
6–<13	31 (58.5%); 22 (41.5%)	53 (100%)
13–18	7 (35%); 13 (65%)	20 (100%)
Total	145 (56.2%); 113 (43.8%)	258 (100%)

n = number of patients; % = percentage within each month (row-wise). The last row (“Total”) shows overall length of admission in the study population.

**Table 6:** Age and complications (*p* = 0.09).

Age Groups: 0–<1, 1–5, 6–<13, 13–18 Years Old			
Age (Years)	No Complications	Complications	Total
0	38 (53.5%)	33 (46.5%)	71 (100%)
1–5	40 (35%)	74 (65%)	114 (100%)
6–<13	20 (37.7%)	33 (62.3%)	53 (100%)
13–18	8 (40%)	12 (60%)	20 (100%)
Total	106 (41%)	152 (59%)	258 (100%)

**Table 7:** Annual number of positive influenza tests, influenza-related hospital admissions, and hospitalization rates among children presenting to the Emergency Department between 2020 and 2025.

Year	Positive Influenza Tests	Influenza-Related Hospital Admissions	Hospitalization Rate (%)
2020	1.464	44	3.01
2021	0	0	N/A
2022	1.697	94	5.54
2023	237	11	4.64
2024	999	50	5.01
2025	1.457	59	4.05
Total	5.854	258	4.41

**Table 8:** Multivariate regression analysis for independent risk factors.

Variable	OR (95% CI)	*p*-Value
Age	1.00	0.920
CRP	1.10	0.076
Oxygen saturation	0.79	0.03
Comorbidity	0.58	0.104

**Table 9:** The characteristics and highlights of Western European and American studies discussing influenza complications in children.

Main Author	Year	Country	Study Type	Main Findings on Complications
Barbieri et al. [19]	2023	Italy	Retrospective cohort (primary care network)	Influenza vaccination rates were low among the studied population. Most common complications: otitis media, pneumonia. No documented deaths.
Dondi et al. [20]	2025	Italy	Retrospective observational study in a tertiary care center	Influenza-associated encephalopathy, lower respiratory tract infections, and myositis were the most common complications. No recorded deaths, but severe complications.
Reinhart et al. [21]	2025	USA	National surveillance	Low vaccination rates, 280 reported influenza-associated deaths, and 3.8 deaths per 1 million children.
Silverman et al. [22]	2025	USA	Multicenter case series	Forty-one cases of influenza-associated acute necrotizing encephalopathy, with high morbidity and mortality rates.
Fazal et al. [23]	2025	USA	CDC-sponsored national surveillance network	The most numerous pediatric influenza-associated deaths since 2004 in the United States of America were noted in the 2024–2025 season, with severe neurological manifestations.

**Table 10:** Characteristics and highlights of Eastern and Central European studies discussing influenza complications in children.

Main Author	Year	Country of Publication and Type of Study	Main Points/Focus
Merișescu, M.M. [15]	2023	Romania—retrospective clinical analysis in tertiary care center	No reported deaths, but 18% of cases were classified as severe. Main complications were respiratory (pneumonia) and hematological (anemia).
Angiel, K. [16]	2025	Poland—retrospective analysis in tertiary care center	No reported admissions to ICU. Most common complications were pneumonia, otitis media, and streptococcal pharyngitis.
Cocuz, M. [17]	2025	Romania—retrospective monocenter study	Study of seasonal influenza in children after the COVID-19 pandemic, emphasizing epidemiological trends and clinical evolution; discusses changes in disease patterns post-pandemic.
Jugulete, G. [18]	2024	Romania—retrospective analysis in a tertiary care center	Describes clinico-epidemiological characteristics of pediatric influenza in the 2023–2024 season; includes data on symptoms, transmission trends, and population impact.

## Data Availability

The datasets used and analyzed in this study are available from the corresponding author on reasonable request. The data are not publicly available due to ethical and privacy-related restrictions.

## Abbreviation

ENT	Ear, Nose, Throat (referring to otolaryngology)
ICU	Intensive Care Unit
CRP	C-reactive protein

## References

[1] Krammer F., Smith G.J.D., Fouchier R.A.M., Peiris M., Kedzierska K., Doherty P.C., Palese P., Shaw M.L., Treanor J., Webster R.G. (2018). Influenza. Nat. Rev. Dis. Primers.

[2] Centers for Disease Control and Prevention (CDC) (2024). Flu and Children.

[3] Unal S., Gökçe M., Aytaç Elmas S., Karabulut E., Altan I., Ozkaya-Parlakay A., Kara A., Ceyhan M., Cengiz A.B., Tuncer M. (2010). Hematological consequences of pandemic influenza H1N1 infection: A single center experience. Turk. J. Pediatr..

[4] Watanabe T., Yoshikawa H., Abe Y., Yamazaki S., Uehara Y., Abe T. (2003). Renal involvement in children with influenza A virus infection. Pediatr. Nephrol..

[5] Tsai J.P., Baker A.J. (2013). Influenza-associated neurological complications. Neurocrit. Care.

[6] American Academy of Pediatrics (2023). Recommendations for prevention and control of influenza in children, 2023–2024. Pediatrics.

[7] Nolan V.G., Arnold S.R., Bramley A.M., Ampofo K., Williams D.J., Grijalva C.G., Self W.H., Anderson E.J., Wunderink R.G., Edwards K.M. (2018). Etiology and impact of coinfections in children hospitalized with community-acquired pneumonia. J. Infect. Dis..

[8] Nickol M.E., Lyle S.M., Dennehy B., Kindrachuk J. (2020). Dysregulated host responses underlie 2009 pandemic influenza-methicillin-resistant *Staphylococcus aureus* coinfection pathogenesis at the alveolar-capillary barrier. Cells.

[9] Rolfes M.A., Foppa I.M., Garg S., Flannery B., Brammer L., Singleton J.A., Burns E., Jernigan D., Olsen S.J., Bresee J. (2018). Annual estimates of the burden of seasonal influenza in the United States: A tool for strengthening influenza surveillance and preparedness. Influ. Other Respir. Viruses.

[10] Tokars J.I., Olsen S.J., Reed C. (2018). Seasonal incidence of symptomatic influenza in the United States. Clin. Infect. Dis..

[11] Lafond K.E., Nair H., Rasooly M.H., Valente F., Booy R., Rahman M., Kitsutani P., Yu H., Guzman G., Coulibaly D. (2016). Global role and burden of influenza in pediatric respiratory hospitalizations, 1982-2012: A systematic analysis. PLoS Med..

[12] Sanz-Muñoz I., Arroyo-Hernantes I., Martín-Toribio A., Toquero-Asensio M., Sánchez-Martínez J., Rodríguez-Crespo C., Eiros J.M. (2025). Disease burden of influenza in Spain: A five-season study (2015–2020). Hum. Vaccin. Immunother..

[13] European Centre for Disease Prevention and Control (2025). Survey Report on National Seasonal Influenza Vaccination Recommendations and Coverage Rates in EU/EEA Countries, 2024/25.

[14] Goldsite Diagnostics Inc. SARS-CoV-2 & Flu A/B & RSV Combo Antigen Test.

[15] Merișescu M.M., Luminos M.L., Pavelescu C., Jugulete G. (2023). Clinical features and outcomes of the association of co-infections in children with laboratory-confirmed influenza during the 2022–2023 season: A Romanian perspective. Viruses.

[16] Angiel K., Stopyra L. (2025). Influenza in hospitalized pediatric patients in Poland: A retrospective single-center study of the 2023/2024 epidemic season. Arch. Med. Sci..

[17] Cocuz M., Dochițoiu M., Cocuz I. (2024). Gripa sezonieră la copii post pandemia de COVID-19-Aspecte epidemiologice și clinico-evolutive. J. Med. Bras..

[18] Jugulete G., Safta M., Gheorghe E., Borcos B., Bajenaru L., Zah L., Negrea D., Popescu A., Merisescu M.M. (2024). Clinico-epidemiological features of influenza in children in the 2023-2024 season. Rom. J. Infect. Dis..

[19] Barbieri E., Porcu G., Donà D., Cavagnis S., Cantarutti L., Scamarcia A., McGovern I., Haag M., Giaquinto C., Cantarutti A. (2023). Epidemiology and burden of influenza in children 0-14 years over ten consecutive seasons in Italy. Pediatr. Infect. Dis. J..

[20] Dondi A., Guida F., Trombetta L., Cocco M.D.P., Piccirilli G., Andreozzi L., Battelli E., Castaldo P., Corsini I., Pierantoni L. (2025). Burden and clinical characteristics of influenza and its complications in children across multiple epidemic seasons. Viruses.

[21] Reinhart K., Huang S., Kniss K., Reed C., Budd A. (2025). Influenza-associated pediatric deaths—United States, 2024–2025influenza season. MMWR Morb. Mortal. Wkly. Rep..

[22] Silverman A., Walsh R., Santoro J.D., Thomas K., Ballinger E., Fisher K.S., Thomas A.X., Appavu B., Kruer M.C., Influenza-Associated Acute Necrotizing Encephalopathy (IA-ANE) Working Group (2025). Influenza-associated acute necrotizing encephalopathy in US children. JAMA.

[23] Fazal A., Harker E.J., Neelam V., Olson S.M., Rolfes M.A., Reinhart K., Kniss K., Frutos A., Leonard J., Reed C. (2025). Pediatric influenza-associated encephalopathy and acute necrotizing encephalopathy—United States, 2024–2025 influenza season. MMWR Morb. Mortal. Wkly. Rep..

[24] Savagner J., Trémeaux P., Baudou E., Mansuy J.M., Cheuret E. (2024). Neurological involvement related to the influenza virus in children: A 5-year single-centre retrospective study. Eur. J. Paediatr. Neurol..

[25] Quertermous B.P., Williams D.J., Bruce J., Sekmen M., Zhu Y., Grijalva C.G., Antoon J.W. (2024). Incidence of influenza-associated neurologic and psychiatric complications requiring hospitalization in children ages 5–17 years. Pediatr. Infect. Dis. J..

[26] Bae S.H., Kim Y.O., Kim S.J., Son Y.J., Woo Y.J. (2010). Transient bulging of the fontanelle in infants after a febrile illness without central nervous system infection. Chonnam Med. J..

[27] Sethuraman C., Holland J., Priego G., Khan F., Johnson R., Keane M. (2023). Bulging anterior fontanelle caused by severe acute respiratory syndrome coronavirus-2. Pediatr. Infect. Dis. J..

[28] Al-Amri M., Alhandi O.H., Ahmad S., Alaido M., Housein F.M., Barkat M.M. (2022). Bulging fontanelle as a sign of COVID-19 infection in infant: A case report. Medicine.

[29] Wolf R.M., Antoon J.W. (2023). Influenza in children and adolescents: Epidemiology, management, and prevention. Pediatr. Rev..

[30] Arranz-Herrero J., Presa J., Rius-Rocabert S., Utrero-Rico A., Arranz-Arija J.Á., Lalueza A., Escribese M.M., Ochando J., Soriano V., Nistal-Villan E. (2023). Determinants of poor clinical outcome in patients with influenza pneumonia: A systematic review and meta-analysis. Int. J. Infect. Dis..

[31] Syridou G., Drikos I., Vintila A., Pegkou A., Zografou L., Roungas P., Papa E., Kyriazopoulos D., Savelieva O., Antonopoulou E. (2019). Influenza A H1N1 associated acute glomerulonephritis in an adolescent. IDCases.

[32] Sellers S.A., Hagan R.S., Hayden F.G. (2017). The hidden burden of influenza: A review of the extra-pulmonary complications of influenza infection. Influ. Other Respir. Viruses.

[33] Watanabe T. (2013). Renal complications of seasonal and pandemic influenza A virus infections. Eur. J. Pediatr..

[34] Kupferman J.C., Trachtman H., Spitzer E.D. (2011). Acute glomerulonephritis and acute kidney injury associated with 2009 influenza A: H1N1 in an infant. Pediatr. Nephrol..

[35] Ashtiani N., Mulder M., Van Wijk A. (2012). A case of tubulointerstitial nephritis in a patient with an influenza H1N1 infection. Pediatr. Nephrol..

[36] Ghiggeri G.M., Losurdo G., Ansaldi F. (2010). Two cases of swine H1N1 influenza presenting with hematuria as prodrome. Pediatr. Nephrol..

[37] Wenderfer S.E. (2015). Viral-associated glomerulopathies in children. Pediatr. Nephrol..

[38] Kerr J., Macartney K., Britton P.N. (2021). Influenza-associated myositis: A single-centre, 5-year retrospective study. Eur. J. Pediatr..

[39] Fretzayas A., Moustaki M., Kotzia D., Nicolaidou P. (2011). Rash, an uncommon but existing feature of H1N1 influenza among children. Influ. Other Respir. Viruses.

[40] Banday A.Z., Arul A., Vignesh P., Singh M.P., Goyal K., Singh S. (2021). Kawasaki disease and influenza-new lessons from old associations. Clin. Rheumatol..

[41] Baigrie D., Crane J.S. (2023). Leukocytoclastic vasculitis. StatPearls.

[42] Cao S., Sun D. (2017). Leucocytoclastic vasculitis following influenza vaccination. BMJ Case Rep..

[43] Lee H.J., Shin D.H., Choi J.S., Kim K.H. (2012). Leukocytoclastic vasculitis associated with influenza A virus infection. J. Korean Med. Sci..

[44] Binns E., Koenraads M., Hristeva L., Flamant A., Baier-Grabner S., Loi M., Lempainen J., Osterheld E., Ramly B., Chakakala-Chaziya J. (2022). Influenza and respiratory syncytial virus during the COVID-19 pandemic: Time for a new paradigm?. Pediatr. Pulmonol..

[45] Cohen R., Ashman M., Taha M.-K., Varon E., Angoulvant F., Levy C., Rybak A., Ouldali N., Guiso N., Grimprel E. (2021). Pediatric Infectious Disease Group (GPIP) position paper on the immune debt of the COVID-19 pandemic in childhood, how can we fill the immunity gap?. Infect. Dis. Now.

[46] Institutul Național de Sănătate Publică (2023). Broșura BT: Gripa 2023.

[47] Villani L., D’Ambrosio F., Ricciardi R., de Waure C., Calabrò G.E. (2022). Seasonal influenza in children: Costs for the health system and society in Europe. Influ. Other Respir. Viruses.

